# IER3 in pancreatic carcinogenesis

**DOI:** 10.18632/oncotarget.4588

**Published:** 2015-06-22

**Authors:** Maria Inés Molejon, Juan Lucio Iovanna

**Affiliations:** Centre de Recherche en Cancérologie de Marseille (CRCM), INSERM U1068, CNRS UMR 7258, Aix-Marseille Université and Institut Paoli-Calmettes, Parc Scientifique et Technologique de Luminy, Marseille, France

**Keywords:** pancreas cancer, PanIN, kras, IER3, ERK1/2

Pancreatic ductal adenocarcinoma (PDAC) has the highest mortality rate of all cancers and the lowest overall survival rate of less than 5% at 5 years. The incidence of PDAC almost equals its mortality rate and is increasing every year, with more than 10,000 predicted new cases in France, 38,000 in the United States and 65,000 in Europe [1].

PDAC is characterized by a stepwise progression of ductal-like cells into malignant adenocarcinoma. This starts with the acinar-ductal metaplasia (ADM), a reprogramming event that induces transdifferentiation to a duct-like phenotype, and results in the pancreatic intraepithelial neoplasia (PanIN) 1A, 1B, 2 and 3 precancerous lesions [2], the latter corresponding to the *in situ* PDAC. The earliest activating mutation occurring in human PDAC is on the GTPase *Kras* oncogene. Previously, we described the importance of the pancreatitis-activated protein NUPR1, which activates RELB during the abovementioned process of transformation [3]. We went on to report that this NUPR1- RELB pathway affects the expression of genes encoding proteins that can serve as effectors of their function in this process, such as the immediate early response 3 (*IER3*) gene defined as essential in PanIN and PDAC development [4].

*IER3* is a stress-inducible gene involved in cellular response to stress. The protein it encodes has multiple actions in signaling pathways associated with the activation of ERK, itself implicating a cascade of phosphorylation events initiated by the stimulation of RAS and leading to phosphorylation of ERK1/2. The ERK-dependent pathway plays a major role in diverse cellular processes, including proliferation, differentiation and survival, and its activity is critical for Kras-dependent PDAC development [5]. Considering that ERK1/2 is mostly inactivated by PP2A, targeting this phosphatase appears an obvious mechanism for regulating Kras oncogenic function. PP2A is a multifarious complex composed of catalytic, scaffold and regulatory subunits which have been divided into three families named PR55, B56 and PR72. In addition, each subunit consists of several members [6].

Using the pancreas as a model, we showed that genetic inactivation of *IER3* in *Kras* oncogene-driven PanIN to PDAC formation leads to a strong delay in PanIN and consequently also in PDAC development. This finding demonstrated, as expected, that *IER3* is an essential contributor to the ADM, PanIN, and PDAC development induced by oncogenic Kras. Mechanistically, we demonstrated that all processes resulting in Nupr1- dependent *IER3* overexpression led to a sustained activation of ERK1/2. Indeed, we showed that IER3 interacts directly with the regulatory subunit of PP2A - B56 - resulting in a sustained phosphorylation of ERK1/2, consequently facilitating the Kras-mediated transformation [4].

Interestingly, *IER3* expression levels, easily detectable during the early transformation processes such as ADM and early PanINs, appeared to decrease or even disappear in late PanIN and PDAC. This would suggest a necessity for *IER3* during early steps of tumor transformation rather than later steps of pancreatic carcinogenesis. This corroborates the Kras-addiction observed during the early but not the later PDAC stages [7]. In this way we demonstrated that *IER3* expression can promote ERK1/2 phosphorylation in PDAC cells but has no influence over tumor growth in xenografts. This explains the inefficiency of anticancer strategies that target ERK1/2 phosphorylation in PDAC treatment.

In conclusion our data indicate that NUPR1 regulates PDAC development through the activation of ERK1/2 phosphorylation in an IER3-dependent manner. IER3 is therefore an important molecule that, by stabilizing ERK1/2 activation, enhances *Kras*-oncogenic effects and thus contributes towards driving PDAC formation. Since *Kras* is found to be mutated in several cancers we consider the possibility that this original molecular mechanism may not be pancreas-specific.

## References

[1] Jemal A (2011). CA: a cancer journal for clinicians.

[2] Almoguera C (1988). Cell.

[3] Hamidi T (2012). The Journal of clinical investigation.

[4] Garcia MN (2014). The Journal of clinical investigation.

[5] Agbunag C (2006). Methods in enzymology.

[6] McCright B (1996). The Journal of biological chemistry.

[7] Lim KH (2005). Cancer cell.

